# Maternal and offspring fasting glucose and type 2 diabetes-associated genetic variants and cognitive function at age 8: a Mendelian randomization study in the Avon Longitudinal Study of Parents and Children

**DOI:** 10.1186/1471-2350-13-90

**Published:** 2012-09-27

**Authors:** Carolina Bonilla, Debbie A Lawlor, Yoav Ben–Shlomo, Andrew R Ness, David Gunnell, Susan M Ring, George Davey Smith, Sarah J Lewis

**Affiliations:** 1 School of Social and Community Medicine, University of Bristol, Bristol, BS8 2BN, United Kingdom; 2 MRC Centre for Causal Analyses in Translational Epidemiology, School of Social and Community Medicine, University of Bristol, Bristol, United Kingdom; 3 School of Oral and Dental Sciences, University of Bristol, Bristol, United Kingdom

**Keywords:** Mendelian randomization, Fasting glucose, Type 2 diabetes, IQ, ALSPAC

## Abstract

**Background:**

In observational epidemiological studies type 2 diabetes (T2D) and both low and high plasma concentrations of fasting glucose have been found to be associated with lower cognitive performance. These associations could be explained by confounding.

**Methods:**

In this study we looked at the association between genetic variants, known to be robustly associated with fasting glucose and T2D risk, in the mother and her offspring to determine whether there is likely to be a causal link between early life exposure to glucose and child’s intelligence quotient (IQ) scores in the Avon Longitudinal Study of Parents and Children (ALSPAC) cohort. We generated a fasting glucose (FGGRS) and a T2D (T2DGRS) genetic risk score and used them in a Mendelian randomization approach.

**Results:**

We found a strong correlation between the FGGRS and fasting glucose plasma measurements that were available for a subset of children, but no association of either the maternal or the offspring FGGRS with child’s IQ was observed. In contrast, the maternal T2DGRS was positively associated with offspring IQ.

**Conclusions:**

Maternal and offspring genetic variants which are associated with glucose levels are not associated with offspring IQ, suggesting that there is unlikely to be a causal link between glucose exposure in utero and IQ in childhood. Further exploration in even larger cohorts is required to exclude the possibility that our null findings were due to a lack of statistical power.

## Background

Brain development occurs most rapidly in utero and in young children up to 2–3 years of age, although it continues well into adulthood [1]. The rapid growth of the foetal and infant brain places an exceptionally high demand on the supply of nutrients from the diet, as adequate levels of certain nutrients are required for cell growth, the synthesis of new tissues, synapse formation and myelination [2]. Glucose is the brain’s main energy source and is transported across the blood–brain barrier, as neurons are unable to store or synthesize it [3,4]. During pregnancy glucose crosses the placenta to meet the energy requirements of the developing foetus since there is almost no foetal gluconeogenesis [5]. High maternal plasma glucose levels, such as those experienced by mothers with poorly controlled diabetes, mean that a greater amount of glucose is transferred to the foetus, which in turn triggers an increased production of foetal insulin and promotes excessive growth in the baby [6]. Children born to mothers with gestational diabetes have been reported to exhibit more inattention and hyperactivity, impaired motor functions, and lower intelligence quotient (IQ) scores and educational attainment [7-9].

In adults, experimental studies where participants’ performance on cognitive tests was evaluated after a meal and a sugary drink, have shown that low plasma glucose levels are associated with poorer results in memory and attention tasks [10,11], as well as in non-memory tasks, especially tasks that are more demanding [3]. Better cognitive function was found to be dependent not only on a higher baseline blood glucose concentration but also on a tighter glucose control (i.e. the ability to keep glucose levels within the normal range) [3]. Elevated glucose levels such as those present in hyperglycaemia and diabetes have also been associated with diminished cognitive function, accelerated cognitive decline and higher risks for developing dementia and Alzheimer’s disease in the elderly [12-14] although not all studies find this [15].

Observational epidemiological studies may be affected by confounding, reverse causation and bias, which makes it more problematic to infer causality [16]. In particular, associations between nutritional status and cognitive ability will be confounded by lifestyle and environmental factors. Ideally, a randomized controlled trial (RCT) would be the way to overcome this problem and obtain evidence for the effects of a modifiable exposure, but, in this case, conducting an RCT would not be ethical. Therefore, we have employed a Mendelian randomization strategy which uses genetic variants that are reliably associated with a modifiable exposure (here, glucose levels) to make inferences about the causal effect of this exposure on an outcome of interest (i.e. IQ scores) [17]. Because genotypes are set randomly at conception and are stable throughout life they are unlikely to be affected by confounders that typically influence observational studies and similarly, will not be affected by reverse causation and bias [17].

Candidate gene and more recently, genomewide association studies (GWAS) have identified a number of genetic variants that are robustly associated with fasting plasma glucose levels [18] and type 2 diabetes (T2D) [19,20]. Together variants associated with fasting glucose explain up to 5% of its genetic variance [21], whereas those associated with T2D account for ~10% of the trait variability [22]. We have genotyped 35 and 38 fasting glucose/T2D-associated variants in the Avon Longitudinal Study of Parents and Children (ALSPAC) mothers and children, respectively, and used them to investigate the relationship between glucose exposure and susceptibility to T2D with child’s IQ at age 8. We examined the association between fasting glucose and IQ using 16 of these variants combined in a fasting glucose genetic risk score (FGGRS) [21]. A type 2 diabetes genetic risk score (T2DGRS) which includes 22 variants was also computed to assess the relationship between T2D risk and IQ test results. The main objective of our study was to establish whether glucose during gestation and childhood, or underlying risk for T2D, are causally related to childhood cognitive ability.

## Methods

### Study population

**Figure 1:**
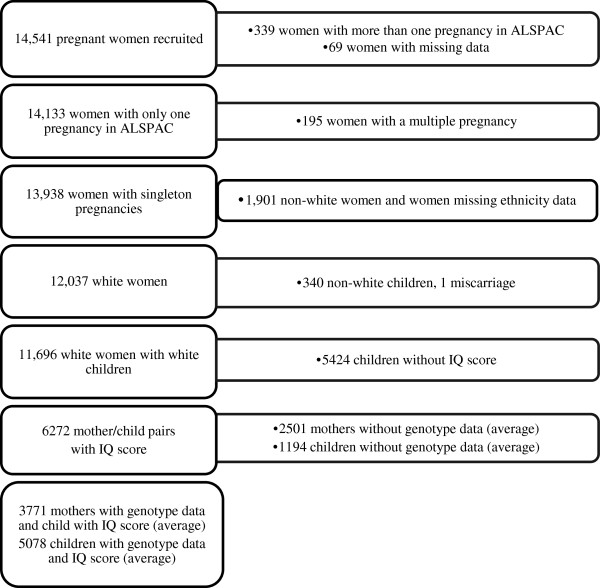
Flow-diagram of participants in the study and reasons for exclusions.

ALSPAC is a population-based prospective cohort study investigating factors that affect the health and development of children and their parents. The study methods are described in detail elsewhere [23,24] ( http://www.alspac.bris.ac.uk). Briefly, pregnant women living around Bristol, England, who had an expected date of delivery between April 1991 and December 1992 were eligible, and of these 14,541 were enrolled in the study. Extensive data have been collected from the mothers and their offspring from pregnancy onwards by questionnaire, abstraction from medical notes, record linkage and by attendance at research clinics. Ethical approval for the study was obtained from the ALSPAC Law and Ethics Committee (IRB# 00003312) and the Local Research Ethics Committees (Bristol and Weston, Southmead, and Frenchay Health Authorities). Written informed consent was obtained from all participants in the study. Numbers of mother-offspring pairs included in the analyses are shown in Figure 1. 

### Assessment of offspring cognitive function

Cognitive testing was carried out by trained psychologists during a clinic visit when the children were aged 8 using a shortened version of the Wechsler Intelligence Scale for Children (WISC-III) consisting of alternate items only (except the coding task) [25]. An overall age adjusted intelligence quotient (IQ) was derived from this assessment for each child who completed the test [26].

### Assessment of fasting glucose and glycosylated hemoglobin (HbA1c) levels in children

From the population cohort a 10% randomly selected sample of parents whose babies were born within the last 6 months of the survey were invited to bring their children to a research clinic (Children in Focus, CIF) at 4, 8 and 12 months of age and 6-monthly intervals thereafter, where a number of clinical, physiological and psychological assessments were carried out. Fasting glucose measurements from children attending the CIF clinic were obtained as part of the Before Breakfast Study done when the children were ~8.5 years of age. Parental consent was sought for venous blood samples, and was given for 81% of the 1135 children in the CIF group. Blood samples were taken after a minimum 6-hour fast. Plasma glucose was measured using a glucose oxidase method implemented on a Y.S.I. model 2300 STAT Plus analyser (Y.S.I., Lynchford House, Farnborough, Hants, UK). Eight-hundred and forty-six children, out of 895 with a measurement of fasting glucose, were included in our study.

At mean age 9 years, 7314 children attended the regular research examination and blood was collected from all children willing to provide a sample. HbA1c was measured from April 2002 to January 2003 from blood obtained during the age 9 years examination using a Menarini HA 8140 auto-analyser which employs a reverse phase cation exchange HPLC with spectrophotometric detection [27]. Results are expressed as a percentage of total hemoglobin. HbA1c measurements were available for 1793 children, 1454 of whom were included in our study.

### Assessment of diabetes categories in mothers

Data on pre-existing diabetes, gestational diabetes and glycosuria was obtained for the index pregnancy from antenatal medical records as reported by Lawlor et al. (2010) [28]. Glycosuria was defined as a measurement of at least 13.9 mmol/l or 250 mg/dl of plasma glucose on at least two occasions at any time during the pregnancy [28]. Women were classified into one of four mutually exclusive categories: no evidence of glycosuria or diabetes, existing diabetes before the pregnancy, gestational diabetes, and glycosuria. A maternal diabetes/glycosuria variable was created and coded as 0 (no diabetes) or 1 (pre-pregnancy diabetes/gestational diabetes/glycosuria).

### Potential confounders

Information on maternal education, social class, age at delivery, parity, interpregnancy interval, smoking, alcohol consumption before and during pregnancy, and iron supplementation was collected using questionnaires completed by the mothers at approximately 18 and 32 weeks of pregnancy. Occurrence of any infection during pregnancy was abstracted from medical records. Children’s date of birth, sex, gestational age and birthweight were obtained from birth records. Breastfeeding duration was recorded from repeat postnatal questionnaires completed by the mother and was categorized as no breastfeeding, breastfeeding for < 3 months, for 3 to 5 months, for ≥ 6 months.

### Genetic variants

DNA was extracted as described previously [29]. Genotyping was undertaken by KBioscience Ltd. ( http://www.kbioscience.co.uk), who use a proprietary competitive allele specific PCR system (KASPar) for SNP analysis. Thirty-five and 38 fasting glucose/T2D-associated SNPs were available for analysis in mothers and children, respectively (Table 1). The top 10 principal components (PCs) that reflect the population’s genetic structure were estimated according to Price et al. [30] from genomewide SNPs genotyped and imputed in ALSPAC mothers and children using Markov Chain Haplotyping software (MACH v.1.0) [31] and phased haplotype data from individuals of European ancestry (CEU) (Hapmap release 22, Phase II NCBI B36, dbSNP 126), based on a cleaned dataset of 8,340 mothers and 8,365 children and 464,311 autosomal SNPs. All 10 PCs were included as covariates in the regression models to account for confounding by population stratification. 

**Table 1:** Genetic variants related to fasting glucose concentration and type 2 diabetes examined in this study

**Gene**	**dbSNP id**	**Alleles (major/minor)**	MAF mothers N = 6844^*^	MAF children N = 7685^*^	**CEU**^**†**^	**Risk allele**^**‡**^	**Reported associations**
*ADAMTS9*	rs4607103	C/T	0.24	0.24	0.19	major	T2D
*ADCY5*	rs2877716	C/T	0.26	0.26	0.25	major	fasting glucose/T2D
*ADIPOQ*	rs1501299	G/T	n/t	0.27	0.27	minor	T2D
*ADIPOQ*	rs17300539	G/A	n/t	0.09	0.08	minor	T2D
*ADIPOQ*	rs266729	C/G	n/t	0.26	0.31	minor	T2D
*ADRA2A*	rs10885122	G/T	0.13	0.12	0.10	major	fasting glucose
*C2CD4B*	rs11071657	A/G	0.39	0.38	0.41	major	fasting glucose
*CDC123/CAMK1D*	rs12779790	A/G	0.18	0.19	0.22	minor	T2D
*CDKAL1*	rs10946398	A/C	0.31	0.31	0.30	minor	T2D
*CDKN2A/B*	rs10811661	T/C	0.18	0.17	0.20	major	T2D
*COX2*	rs20417	G/C	0.15	0.15	0.18	major	T2D
*CRY2*	rs11605924	C/A	0.48	0.48	0.54	minor	fasting glucose
*DGKB/TMEM195*	rs2191349	T/G	0.46	0.46	0.53	major	fasting glucose/T2D
*FADS1*	rs174550	T/C	0.33	0.33	0.37	major	fasting glucose
*FTO*	rs9939609	T/A	0.40	0.40	0.45	minor	obesity
*G6PC2*	rs560887	C/T	0.30	0.30	0.33	major	fasting glucose
*GCK*	rs1799884	G/A	0.18	0.18	0.20	minor	fasting glucose/T2D
*GCKR*	rs780094	C/T	0.39	0.39	0.38	major	fasting glucose/T2D
*GLIS3*	rs7034200	C/A	0.47	0.48	0.53	minor	fasting glucose
*HHEX-IDE*	rs1111875	C/T	0.41	0.41	0.44	major	T2D
*HNF1B*	rs757210	C/T	0.37	0.37	0.43	minor	T2D
*IGF2BP2*	rs4402690	G/T	0.31	0.30	0.29	minor	T2D
*JAZF1*	rs864745	A/G	0.50	0.50	0.48	major	T2D
*KCNJ11*	rs5219	C/T	0.35	0.35	0.50	minor	T2D
*KCNQ1*	rs2237892	C/T	0.06	0.06	0.08	major	T2D
*KCNQ1*	rs2237895	A/C	0.42	0.41	0.36	minor	T2D
*MADD*	rs7944584	A/T	0.28	0.28	0.29	major	fasting glucose
*MTNR1B*	rs10830963	C/G	0.27	0.28	0.30	minor	fasting glucose/T2D
*NOTCH2*	rs10923931	G/T	0.11	0.11	0.09	minor	T2D
*PPARG*	rs1801282	C/G	0.12	0.11	0.07	major	T2D
*PROX1*	rs340874	C/T	0.43	0.43	0.44	major	fasting glucose/T2D
*SLC2A2*	rs11920090	T/A	0.12	0.13	0.13	major	fasting glucose
*SLC30A8*	rs13266634	C/T	0.30	0.30	0.25	major	fasting glucose/T2D
*TCF7L2*	rs12255372	G/T	0.29	0.29	0.22	minor	fasting glucose
*TCF7L2*	rs7903146	C/T	0.30	0.30	0.25	minor	fasting glucose/T2D
*THADA*	rs7578597	A/G	0.11	0.11	0.08	major	T2D
*TSPAN8/LGR5*	rs7961581	T/C	0.28	0.28	0.23	minor	T2D
*WFS1*	rs10010131	G/A	0.40	0.40	0.26	major	T2D

*MAF* = minor allele frequency.

*T2D* = type 2 diabetes.

*n/t* = not typed.

^*^N = mean of the total number of individuals typed for each SNP. Range in mothers: 2553–7151. Range in children: 7295–8323.

^**†**^minor allele frequency in the HapMap CEPH European population.

^**‡**^risk allele for higher plasma fasting glucose or susceptibility to type 2 diabetes.

### Genetic risk scores

We computed a weighted fasting glucose genetic risk score (FGGRS) as described by Barker et al. [21] using 16 genetic variants –out of the set of 35/38- that had been reliably associated with fasting glucose levels in prior studies [18]. Each SNP genotype was coded as 0, 1 or 2 depending on the number of risk alleles the individual carries, and their effects on fasting glucose levels in adults were obtained from Barker et al. [21]. For each individual FGGRS was calculated as follows:

 FGGRS = [(0.075*rs560887 + 0.067*rs10830963 + 0.062* rs1799884 + 0.03*rs2191349 + 0.029*rs780094 + 0.027*rs2877716 + 0.027*rs13266634 + 0.023*rs7903146 + 0.022*rs10885122 + 0.021*rs7944584 + 0.02*rs11920090 + 0.018*rs7034200 + 0.017*rs174550 + 0.015*rs11605924 + 0.013*rs340874 + 0.008*rs11071657)/0.474]*16, where the denominator corresponds to the total effect size (i.e. sum of all SNP effects).

Two SNPs tested in this study were different from those used by Barker et al., but they were located in the same genes and were in strong linkage disequilibrium: rs1799884 in *GCK* instead of rs4607517 (r^2^ = 1.0), and rs2877716 in *ADCY5* instead of rs11708067 (r^2^ = 0.8), therefore the same effect was assumed.

Similarly, we calculated a type 2 diabetes genetic risk score (T2DGRS) made up of the 22 variants from the 35/38 set that have been previously associated with the disease. Excluded variants and reasons for their exclusion were: all three SNPs in *ADIPOQ* because they had been typed only in children, rs12779790 in *CDC123/CAMK1D* had a considerably lower sample size in mothers, and rs20417 in *COX2* because its effects have only been estimated in Pima Indians [32]. Eight SNPs were common to both genetic scores. Effects for polymorphisms in the T2DGRS were obtained from Hivert et al. [33] and the GWAS catalog. As with the FGGRS, effects reported for rs917793 and rs11708067 were used in place of those corresponding to the linked SNPs rs1799884 and rs2877716. The effect of SNP rs2237895 was obtained from Unoki et al. (2008) [34]. The T2DGRS was computed as:

T2DGRS = [(0.09*rs4607103 + 0.15*rs10946398 + 0.23*rs10811661 + 0.16*rs1111875 + 0.1*rs757210 + 0.16*rs4402960 + 0.1*rs864745 + 0.15*rs5219 + 0.22*rs2237895 + 0.12*rs10923931 + 0.17*rs1801282 + 0.14*rs7578597 + 0.09*rs7961581 + 0.1*rs10010131 + 0.11*rs2877716 + 0.06*rs2191349 + 0.07*rs1799884 + 0.06*rs780094 + 0.09*rs10830963 + 0.07*rs340874 + 0.14*rs13266634+ 0.31*rs7903146)/2.86]*22.

In both FGGRS and T2DGRS, variants in strong linkage disequilibrium with others already in the score were left out as well (i.e. *TCF7L2* rs12255372 and *KCNQ1* rs2237892). From each pair of linked variants we selected the variant with the greatest effect or if effects were similar, the best studied variant in European populations.

### Statistical analysis

Maternal and offspring genotypes were checked for deviation from Hardy-Weinberg equilibrium using the hwsnp function implemented in the program Stata 11 (Stata Corporation, College Station, Texas). We investigated the association of potential confounding factors with exposures (i.e. fasting glucose and HbA1c concentrations, maternal diabetes/glycosuria, maternal and offspring genotypes and genetic risk scores) and outcome (offspring IQ), as well as that of exposures and outcome using linear regression and chi square tests as appropriate. The association of offspring fasting glucose and HbA1c levels with IQ was adjusted for child’s age and sex, whereas that of maternal diabetes/glycosuria with IQ was additionally adjusted for maternal age at delivery, birthweight, gestational age and duration of breastfeeding. As a child’s genotype may influence childhood plasma fasting glucose, we controlled for it to specifically assess the effect on IQ of maternal fasting glucose status during pregnancy. Single variant analysis was performed assuming an additive genetic model with the major allele homozygote genotype as baseline. All analyses involving genotypes or genetic risk scores and IQ were also adjusted for the top 10 PCs as markers of population stratification. The statistical package Stata 11 was used to carry out all tests.

## Results

WISC scores at age 8 were available for 6272 children (49.9% of whom were male). The mean IQ score was 104.4 (SD, 16.4), assessed at 8 years 7 months (SD, 3.3 months) on average. The majority of ALSPAC children exhibited fasting glucose and HbA1c levels within the reference range (mean ± SD fasting glucose: 4.95 ± 0.39 mmol/l; mean ± SD HbA1c: 4.91 ± 0.31%). According to the World Health Organization diabetes mellitus is diagnosed when fasting plasma glucose is ≥7.0 mmol/l or HbA1c is ≥ 6.5% [35,36]. Three ALSPAC children had fasting glucose levels compatible with a diabetes diagnosis, the same was true for HbA1c levels in two other children.

The number of individuals included in the main analysis of genotype and IQ are shown in Figure 1. Table 1 lists all the genetic variants selected for the study, establishing whether they have been earlier associated with fasting glucose or T2D or both.

Three SNPs in the mothers (rs10923931, rs757210, rs340874) and two in the children (rs757210, rs340874) were out of Hardy-Weinberg equilibrium (p ≤ 0.02) although this is not the case if a Bonferroni correction for multiple testing is applied. These SNPs were not excluded from the analysis. Allele frequencies for these variants were similar to those reported for the HapMap CEU population (Table 1).

### Confounders

Offspring IQ was associated with 13 out of 15 (87%) of the covariables considered here at a nominal p-value threshold of 0.05. Fasting glucose in children was associated with sex, with girls showing lower levels of plasma fasting glucose than boys (mean ± SD: 4.88 ± 0.38 vs 5.01 ± 0.40). Both fasting glucose and HbA1c were associated with duration of breastfeeding. Children breastfed for three months or less exhibited the highest mean difference in fasting glucose (0.07 mmol/l; 95% CI −0.02, 0.15) and HbA1c (0.10%; 95% CI 0.05, 0.15) levels with respect to never breastfed children. Maternal diabetes/glycosuria was strongly associated with gestational age (mean difference in gestational age between diabetic and non-diabetic mothers: -0.28 weeks; 95% CI −0.45, -0.12), birthweight (mean difference in child’s birthweight between diabetic and non-diabetic mothers: 96.5 g; 95% CI 45.7, 147.2) and duration of breastfeeding (34% of diabetic mothers never breastfed compared to 25% of non-diabetic mothers).

In children, 29 associations of genotype with confounders were expected below a nominal p-value of 0.05 in a total of 570 tests (15 confounders x 38 SNPs) and 43 (7.5%) were observed. Whereas among mothers 26 associations were expected and 28 (5.3%) were found in 525 tests (15 confounders x 35 SNPs). Both maternal risk scores were associated with gestational age, and T2DGRS was also associated with child’s sex. Offspring FGGRS and T2DGRS were both associated with interpregnancy interval and alcohol intake. Whereas FGGRS was also associated with taking iron supplementation during pregnancy, and T2DGRS was associated with smoking and maternal age at delivery as well (data available on request). However, the associations of genetic variants and scores with confounders were not strong enough to overcome a Bonferroni correction for multiple testing.

In summary, while we detected strong associations of confounders with the outcome (i.e. IQ) and exposures (i.e. fasting glucose and HbA1c levels, maternal diabetes/glycosuria) of interest, that was not the case with the genetic variants and scores.

### Association of SNPs and genetic risk scores with fasting glucose and HbA1c levels in children

The offspring FGGRS followed a normal distribution, with a mean score of 17.1 and a range between 6.5 and 26.9. It was strongly and positively associated with offspring fasting glucose and HbA1c levels, explaining 2-3% of trait variance, which indicates that it may be able to tell us something about the effect of glucose on outcome (Table 2). Adjustment for population stratification did not alter the estimated effect (Table 2).

**Table 2:** Mean difference in fasting glucose and HbA1c levels per weighted allele of genetic risk score in children

**Genetic risk score**	**N**	**Fasting glucose (mmol/l)**	**p-value**	**N**	**Adjusted by PCs**	**p-value**
FGGRS	573	0.02 (0.01, 0.03)	7.1x10^-5^	522	0.02 (0.01, 0.03)	0.001
T2DGRS	511	0.003 (−0.01, 0.01)	0.59	467	0.003 (−0.01, 0.01)	0.61
	**N**	**HbA1c (%)**	**p-value**	**N**	**Adjusted by PCs**	**p-value**
FGGRS	1037	0.02 (0.01, 0.02)	1.6x10^-6^	933	0.02 (0.01, 0.02)	1.4x10^-5^
T2DGRS	918	0.004 (−0.002, 0.01)	0.20	831	0.005 (−0.002, 0.01)	0.19

*FGGRS* = fasting glucose genetic risk score.

*T2DGRS* = type 2 diabetes genetic risk score.

*PCs* = population stratification principal components.

Likewise, the T2DGRS in children was distributed normally, with a mean of 22.6 (range: 8.9-33.9). No association was detected with fasting glucose and HbA1c plasma concentrations (Table 2).

Individual SNPs in the genes *ADCY5, G6PC2, GCK* and *HNF1B* were associated with fasting glucose levels (see Additional file 1: Table S1). With the exception of rs757210 in *HNF1B*, which has been linked only to T2D, the remaining three SNPs had been previously associated with fasting glucose [18]. Individual SNPs associated with HbA1c were located in *MTNR1B*, *SLC2A2*, *G6PC2*, *WFS1* and *GCKR*. All variants were reported to be associated with fasting glucose although rs10010131 in *WFS1* was associated with fasting glucose and HbA1c levels only among individuals who developed hyperglycaemia or T2D [18,37] (see Additional file 2: Table S2).

### Association of SNPs and genetic risk scores with maternal diabetes/glycosuria

We did not have fasting glucose and HbA1c plasma levels in pregnancy for the mothers to perform a similar validation assessment. Although we were aware of limited statistical power, we did explore the association of maternal genetic risk scores and single variants with the maternal diabetes/glycosuria phenotype. The FGGRS was not associated with this variable but the T2DGRS showed a marginal association (mean difference in T2DGRS between diabetic and non-diabetic mothers: 0.48; 95% CI, -0.01, 0.98; p = 0.06). When SNPs were tested individually we found evidence of association with rs7903146 in *TCF7L2* and rs10830963 in *MTNR1B*.

### Association of fasting glucose concentration, HbA1c fraction, SNPs and genetic risk scores with IQ in children

Fasting glucose was inversely associated with IQ scores although the effect was small and imprecisely estimated. HbA1c levels were not associated with IQ. The FGGRS also appeared to be inversely associated with IQ but again this is not distinguishable from the null hypothesis. Equally, no association was found between the T2DGRS and child’s IQ (Table 3).

**Table 3:** Association of children’s fasting glucose, HbA1c levels, and genetic risk scores, with IQ at age 8

**Exposure**	**N (unadjusted/adjusted)**	**Mean difference in IQ per unit change in exposure (95% CI)**	**p-value**	**Adjusted by child’s age and sex**	**p-value**
fasting glucose (mmol/l)	784/784	−1.94 (−4.71, 0.82)	0.17	−2.22 (−5.01, 0.56)	0.12
HbA1c (%)	1198/1198	0.43 (−2.53, 3.40)	0.78	0.65 (−2.29, 3.59)	0.67
**Genetic risk score**	**N (unadjusted/adjusted)**	**Mean difference in IQ per weighted allele (95% CI)**	**p-value**	**Adjusted by PCs**	**p-value**
FGGRS	3904/3457	−0.09 (−0.26, 0.08)	0.29	−0.11 (−0.30, 0.07)	0.22
T2DGRS	3468/3053	0.01 (−0.15, 0.18)	0.87	0.06 (−0.12, 0.24)	0.52

*FGGRS* = fasting glucose genetic risk score.

*T2DGRS* = type 2 diabetes genetic risk score.

*PCs* = population stratification principal components.

When variants were analyzed independently, associations between SNPs rs10811661 in *CDKN2A/2B*, rs1801282 in *PPARG* and rs7961581 in *TSPAN8-LGR5*, and IQ were identified (see Additional file 3: Table S3). After adjustment for population stratification SNPs rs10811661, rs1801282, rs20417 in *COX2*, rs2237892 in *KCNQ1* show evidence for association with IQ (see Additional file 4: Table S4). These variants have been previously related to T2D risk but not to fasting glucose (Table 1) [19,20,32,38]. In the first two cases the allele that increases risk for developing T2D was associated with a higher IQ score while the opposite was true for *COX2* and *KCNQ1*.

### Association of maternal SNPs and genetic risk scores with offspring IQ

Maternal FGGRS was normally distributed, with a mean score of 17.1 and a range between 6.8 and 27.6. There was no evidence of an association with offpring IQ (Table 4). The maternal T2DGRS exhibited a normal distribution with mean 22.6 and a range between 10.9 and 34.7, but, unlike the FGGRS, there was some evidence that it was positively associated with offspring IQ after adjustment for child’s T2DGRS and population stratification (Table 4).

**Table 4:** Association of maternal genetic risk scores with offspring IQ at age 8

**Genetic risk score**	**N (unadjusted/adjusted)**	**Mean difference in IQ per weighted allele (95% CI)**	**p-value**	**Adjusted by offspring score and PCs**	**p-value**
FGGRS	2732/1374	−0.03 (−0.24, 0.17)	0.74	0.20 (−0.14, 0.53)	0.26
T2DGRS	2423/1102	0.16 (−0.04, 0.36)	0.12	0.47 (0.12, 0.81)	0.01

*FGGRS* = fasting glucose genetic risk score.

*T2DGRS* = type 2 diabetes genetic risk score.

*PCs* = population stratification principal components.

In the single variant analysis, SNPs rs7034200 in *GLIS3*, rs864745 in *JAZF1* and rs340874 in *PROX1* were associated with child’s IQ (see Additional file 5: Table S5). Adjustment for offspring genotype and population stratification showed additional associations of rs560887 in *G6PC2* and rs10010131 in *WFS1*, but the association with *GLIS3* was no longer evident (see Additional file 6: Table S6). In *JAZF1* and *G6PC2* the allele associated with susceptibility to T2D or higher plasma fasting glucose was associated with a higher IQ, whilst in *PROX1* and *WFS1* the risk allele appeared to reduce IQ.

### Association of maternal diabetes/glycosuria with offpring IQ

Offspring of mothers with diabetes/glycosuria exhibited a lower IQ score than children born to non-diabetic mothers (mean difference in offspring IQ between diabetic and non-diabetic mothers: -3.5, 95% CI −5.6, -1.5; p = 0.001; with adjustment for child’s age and sex, maternal age at delivery, birthweight, gestational age and duration of breastfeeding).

## Discussion

We have confirmed that a FGGRS is strongly associated with fasting glucose in a population based cohort and therefore may be used to determine the effect of glucose exposure on a phenotype of interest. It was not only strongly associated with fasting glucose levels but also with levels of HbA1c [39]. Not all SNPs reported to be associated with fasting glucose [18] showed an association with this exposure in our analysis but three of the variants with the strongest effects in earlier studies did (i.e. those in *ADCY5*, *G6PC2* and *GCK*). Genes associated with HbA1c in our study include two described in a previous GWAS of HbA1c (*MTNR1B* and *G6PC2*), although the variants examined were different [39], and a SNP in *WFS* reportedly linked to HbA1c in a prospective epidemiological study conducted in France [37].

The T2DGRS, including all variants associated with T2D, some of which were also associated with fasting glucose, was not associated with plasma fasting glucose or HbA1c. Neither were the maternal and the offspring FGGRS nor the offspring T2DGRS associated with IQ in the ALSPAC population. In contrast, the maternal T2DGRS was associated with an increase of 0.03SD in child’s IQ after adjustment for offspring T2DGRS and population stratification.

Additionally, we uncovered associations with IQ for a few individual SNPs in mothers and children, the majority of which were T2D but not fasting glucose-associated variants. Only the variants in *PROX1* and *G6PC2* had been associated with levels of fasting glucose before [18,40]. Since there was some indication that the *PROX1* SNP was in HW disequilibrium in mothers and children this association should be carefully assessed. However, evidence for the association of IQ with individual variants disappears after a Bonferroni correction for multiple testing (cut-off p-value: 0.001).

We did not detect an association of the FGGRS with diabetes/glycosuria in the mothers, yet this outcome was positively associated with the T2DGRS. SNPs independently associated with maternal diabetes/glycosuria had been earlier related to plasma fasting glucose regulation and T2D risk.

Overall, there was very little overlap between the SNPs associated with fasting glucose and HbA1c levels, maternal diabetes/glycosuria and IQ scores in ALSPAC.

Given the results described above, we have not found sufficient evidence that fasting glucose concentration either in mothers during gestation or in children later in life affects cognitive ability at mean age 8, but we lacked statistical power to reliably establish the absence of association. Despite the FGGRS being strongly associated with fasting glucose in plasma its effects are quite small therefore a larger sample size will be required to carry out an adequately powered analysis.

On the other hand, we have uncovered some evidence that children of mothers who carry T2D susceptibility alleles exhibit higher IQ scores than children of non-carriers. This appears to be in disagreement with recent studies, and our study as well, that have revealed that maternal pre-pregnancy diabetes and gestational diabetes, especially with poor metabolic control, have an adverse effect on offspring cognition [9,41,42], although not all studies have detected a reduction in IQ scores [43,44]. We speculate that the presence of T2D risk alleles (which could be contributing to maintain higher glucose levels in blood) may be beneficial during pregnancy as women have to fulfil the baby’s glucose requirements besides their own. However, having a fully established disease phenotype, such as gestational diabetes, can in turn be detrimental, either because glucose levels end up being excessively high or because of the existence of factors related to the disease, other than hyperglycemia, that unfavourably influence neurodevelopment and are not captured by these genetic variants and scores. Additionally, the negative association between diabetes and cognition could be the result of confounding by lifestyle and other factors such as poor diet, smoking, alcohol intake and a lower educational achievement. Further studies are definitely needed to examine this issue in greater detail.

### Strengths and limitations

Among the strengths of our study is having access to a large birth cohort such as ALSPAC to explore the role of maternal fasting glucose status during pregnancy and their offspring own fasting glucose levels in relation to cognitive ability in children. Then, the validation in the ALSPAC population of a robust instrument such as the FGGRS, which can be used in future Mendelian randomization studies to determine the causal relationship between fasting glucose (or HbA1c) levels and specific phenotypes. Lack of power to obtain conclusive results is likely the major weakness of this study. Additionally, because most individuals had fasting glucose and HbA1c levels within the normal range, it may have not been possible in this setting to unravel an association that manifests itself only under very high or very low glucose concentrations.

## Conclusions

Based on our results we conclude that genetic variants associated with subtle variations in fasting glucose levels do not have an important influence on IQ. However, additional studies in larger cohorts should be carried out to confirm these findings.

SJL, GDS, DAL, DG, YBS, ARN designed the study; SMR oversaw the laboratory analyses; CB analyzed the data; CB, SJL wrote the first draft of the manuscript; all authors critically reviewed the manuscript and approved the final version.

## Abbreviations

ALSPAC: Avon longitudinal study of parents and children; FGGRS: Fasting glucose genetic risk score; GWAS: Genomewide association study; IQ: Intelligence quotient; RCT: Randomized controlled trial; SNP: Single nucleotide polymorphism; T2D: Type 2 diabetes; T2DGRS: Type 2 diabetes genetic risk Score; WISC: Wechsler intelligence scale for children.

## Competing interests

The authors declare that they have no competing interests.

## Authors’ contributions

SJL, GDS, DAL, DG, YBS, ARN designed the study; SMR oversaw the laboratory analyses; CB analysed the data; CB, SJL wrote the first draft of the manuscript; all authors critically reviewed the manuscript and approved the final version.

## Pre-publication history

The pre-publication history for this paper can be accessed here:

http://www.biomedcentral.com/1471-2350/13/90/prepub

## Supplementary Material

Additional file 1 **Table S1. **Association of SNPs in fasting glucose and type 2 diabetes-related genes with fasting glucose levels in children. Click here for file

Additional file 2 **Table S2. **Association of SNPs in fasting glucose and type 2 diabetes-related genes with HbA1c levels in children. Click here for file

Additional file 3 **Table S3. **Association of SNPs in fasting glucose and type 2 diabetes-related genes in children with IQ at age 8. Click here for file

Additional file 4 **Table S4. **Association of SNPs in fasting glucose and type 2 diabetes-related genes in children with IQ at age 8, adjusted for population stratification. Click here for file

Additional file 5 **Table S5. **Association of maternal SNPs in fasting glucose and type 2 diabetes-related genes with offspring IQ at age 8. Click here for file

Additional file 6 **Table S6. **Association of maternal SNPs in fasting glucose and type 2 diabetes-related genes with offspring IQ at age 8, adjusted for offspring genotype and population stratification. Click here for file
