# Supplementary material for: Maternal and offspring fasting glucose and type 2 diabetes-associated genetic variants and cognitive function at age 8: a Mendelian randomization study in the Avon Longitudinal Study of Parents and Children
Source: BMC Med Genet. 2012 Sep 27;13:90. doi: 10.1186/1471-2350-13-90 (PMC3570299; doi:10.1186/1471-2350-13-90)
Supplement: Additional file 1 — Table S1. Association of SNPs in fasting glucose and type 2 diabetes-related genes with fasting glucose levels in children. [file 1471-2350-13-90-S1.doc]

**Additional Table 1.** Association of SNPs in fasting glucose and type 2 diabetes-related genes with fasting glucose levels in children.

| **gene** | **dbSNP id** | **mean difference in fasting glucose per minor allele**  **(95% CI)** | **p-value** | **N** |
| --- | --- | --- | --- | --- |
| *ADAMTS9* | rs4607103 | 0.004 (-0.04, 0.05) | 0.86 | 720 |
| *ADCY5* | rs2877716 | -0.05 (-0.09, -0.002) | 0.04 | 715 |
| *ADIPOQ* | rs1501399 | 0.03 (-0.02, 0.07) | 0.28 | 730 |
| *ADIPOQ* | rs17300539 | -0.01 (-0.08, 0.06) | 0.77 | 722 |
| *ADIPOQ* | rs266729 | -0.04 (-0.08, 0.01) | 0.12 | 728 |
| *ADRA2A* | rs10885122 | -0.01 (-0.07, 0.04) | 0.64 | 818 |
| *C2CD4B* | rs11071657 | -0.02 (-0.05, 0.02) | 0.32 | 821 |
| *CDC123/CAMK1D* | rs12779790 | 0.04 (-0.01, 0.10) | 0.10 | 726 |
| *CDKAL1* | rs10946398 | 0.04 (-0.01, 0.08) | 0.08 | 719 |
| *CDKN2A/B* | rs10811661 | 0.01 (-0.04, 0.06) | 0.66 | 728 |
| *COX2* | rs20417 | 0.05 (-0.004, 0.10) | 0.07 | 811 |
| *CRY2* | rs1160592 | 0.002 (-0.04, 0.04) | 0.92 | 825 |
| *DGKB/TMEM195* | rs2191349 | 0.002 (-0.04, 0.04) | 0.93 | 725 |
| *FADS1* | rs174550 | -0.02 (-0.06, 0.02) | 0.38 | 822 |
| *FTO* | rs9939609 | -0.02 (-0.06, 0.03) | 0.42 | 694 |
| *G6PC2* | rs560887 | -0.05 (-0.10, -0.01) | 0.02 | 721 |
| *GCK* | rs1799884 | 0.09 (0.04, 0.15) | 0.001 | 738 |
| *GCKR* | rs780094 | -0.04 (-0.08, 0.01) | 0.09 | 725 |
| *GLIS3* | rs7034200 | -0.008 (-0.05, 0.03) | 0.67 | 817 |
| *HHEX-IDE* | rs1111875 | -0.03 (-0.08, 0.01) | 0.12 | 728 |
| *HNFB1* | rs757210 | -0.05 (-0.09, -0.01) | 0.02 | 722 |
| *IGF2BP2* | rs4402690 | 0.01 (-0.03, 0.06) | 0.66 | 729 |
| *JAZF1* | rs864745 | 0.02 (-0.03, 0.06) | 0.43 | 720 |
| *KCNJ11* | rs5219 | -0.02 (-0.06, 0.02) | 0.43 | 727 |
| *KCNQ1* | rs2237892 | 0.05 (-0.03, 0.14) | 0.21 | 733 |
| *KCNQ1* | rs2237895 | -0.01 (-0.06, 0.03) | 0.50 | 726 |
| *MADD* | rs7944584 | 0.001 (-0.04, 0.04) | 0.98 | 823 |
| *MTNR1B* | rs10830963 | 0.03 (-0.02, 0.08) | 0.22 | 731 |
| *NOTCH2* | rs10923931 | 0.04 (-0.03, 0.10) | 0.27 | 727 |
| *PPARG* | rs1801282 | 0.004 (-0.06, 0.07) | 0.90 | 717 |
| *PROX1* | rs340874 | 0.02 (-0.02, 0.06) | 0.28 | 816 |
| *SLC2A2* | rs11920090 | -0.04 (-0.09, 0.02) | 0.20 | 824 |
| *SLC30A8* | rs13266634 | -0.02 (-0.07, 0.02) | 0.34 | 723 |
| *TCF7L2* | rs12255372 | 0.001 (-0.04, 0.04) | 0.98 | 744 |
| *TCF7L2* | rs7903146 | 0.01 (-0.04, 0.05) | 0.76 | 707 |
| *THADA* | rs7578597 | -0.02 (-0.08, 0.04) | 0.51 | 729 |
| *TSPAN8/LGR5* | rs7961581 | -0.03 (-0.08, 0.01) | 0.18 | 722 |
| *WFS1* | rs10010131 | -0.04 (-0.08, 0.003) | 0.07 | 731 |
