# Supplementary material for: Maternal and offspring fasting glucose and type 2 diabetes-associated genetic variants and cognitive function at age 8: a Mendelian randomization study in the Avon Longitudinal Study of Parents and Children
Source: BMC Med Genet. 2012 Sep 27;13:90. doi: 10.1186/1471-2350-13-90 (PMC3570299; doi:10.1186/1471-2350-13-90)
Supplement: Additional file 2 — Table S2. Association of SNPs in fasting glucose and type 2 diabetes-related genes with HbA1c levels in children. [file 1471-2350-13-90-S2.doc]

**Additional Table 2.** Association of SNPs in fasting glucose and type 2 diabetes-related genes with HbA1c levels in children.

| **gene** | **dbSNP id** | **mean difference in HbA1c**  **per minor allele (95% CI)** | **p-value** | **N** |
| --- | --- | --- | --- | --- |
| *ADAMTS9* | rs4607103 | -0.0003 (-0.03, 0.03) | 0.98 | 1289 |
| *ADCY5* | rs2877716 | -0.02 (-0.05, 0.003) | 0.08 | 1266 |
| *ADIPOQ* | rs1501399 | 0.01 (-0.02, 0.03) | 0.59 | 1296 |
| *ADIPOQ* | rs17300539 | 0.03 (-0.01, 0.07) | 0.18 | 1290 |
| *ADIPOQ* | rs266729 | -0.02 (-0.04, 0.01) | 0.26 | 1304 |
| *ADRA2A* | rs10885122 | -0.03 (-0.06, 0.01) | 0.12 | 1435 |
| *C2CD4B* | rs11071657 | 0.01 (-0.02, 0.03) | 0.63 | 1442 |
| *CDC123/CAMK1D* | rs12779790 | 0.02 (-0.01, 0.05) | 0.18 | 1288 |
| *CDKAL1* | rs10946398 | 0.02 (-0.004, 0.05) | 0.09 | 1286 |
| *CDKN2A/B* | rs10811661 | -0.02 (-0.05, 0.02) | 0.33 | 1302 |
| *COX2* | rs20417 | -0.01 (-0.04, 0.02) | 0.68 | 1425 |
| *CRY2* | rs1160592 | -0.02 (-0.04, 0.004) | 0.11 | 1442 |
| *DGKB/TMEM195* | rs2191349 | 0.01 (-0.01, 0.04) | 0.31 | 1293 |
| *FADS1* | rs174550 | -0.01 (-0.04, 0.01) | 0.24 | 1437 |
| *FTO* | rs9939609 | -0.01 (-0.04, 0.01) | 0.36 | 1273 |
| *G6PC2* | rs560887 | -0.03 (-0.06, -0.01) | 0.01 | 1292 |
| *GCK* | rs1799884 | 0.03 (-0.0003, 0.06) | 0.05 | 1331 |
| *GCKR* | rs780094 | -0.03 (-0.05, -0.003) | 0.03 | 1300 |
| *GLIS3* | rs7034200 | -0.005 (-0.03, 0.02) | 0.65 | 1431 |
| *HHEX-IDE* | rs1111875 | -0.01 (-0.04, 0.01) | 0.32 | 1297 |
| *HNFB1* | rs757210 | -0.0005 (-0.02, 0.02) | 0.97 | 1288 |
| *IGF2BP2* | rs4402690 | -0.01 (-0.03, 0.02) | 0.59 | 1302 |
| *JAZF1* | rs864745 | 0.01 (-0.01, 0.03) | 0.44 | 1282 |
| *KCNJ11* | rs5219 | 0.002 (-0.02, 0.03) | 0.88 | 1292 |
| *KCNQ1* | rs2237892 | 0.002 (-0.05, 0.05) | 0.93 | 1288 |
| *KCNQ1* | rs2237895 | -0.001 (-0.03, 0.02) | 0.91 | 1288 |
| *MADD* | rs7944584 | -0.01 (-0.03, 0.02) | 0.48 | 1437 |
| *MTNR1B* | rs10830963 | 0.05 (0.02, 0.08) | 2.8x10^-4^ | 1305 |
| *NOTCH2* | rs10923931 | -0.02 (-0.06, 0.01) | 0.22 | 1293 |
| *PPARG* | rs1801282 | 0.004 (-0.03, 0.04) | 0.84 | 1289 |
| *PROX1* | rs340874 | 0.01 (-0.01, 0.04) | 0.23 | 1436 |
| *SLC2A2* | rs11920090 | -0.04 (-0.07, -0.01) | 0.01 | 1442 |
| *SLC30A8* | rs13266634 | 0.003 (-0.02, 0.03) | 0.81 | 1290 |
| *TCF7L2* | rs12255372 | 0.002 (-0.02, 0.03) | 0.87 | 1320 |
| *TCF7L2* | rs7903146 | 0.002 (-0.02, 0.03) | 0.88 | 1262 |
| *THADA* | rs7578597 | -0.03 (-0.06, 0.01) | 0.15 | 1294 |
| *TSPAN8/LGR5* | rs7961581 | -0.01 (-0.03, 0.02) | 0.61 | 1293 |
| *WFS1* | rs10010131 | 0.03 (0.01, 0.05) | 0.02 | 1299 |
