# Supplementary material for: Maternal and offspring fasting glucose and type 2 diabetes-associated genetic variants and cognitive function at age 8: a Mendelian randomization study in the Avon Longitudinal Study of Parents and Children
Source: BMC Med Genet. 2012 Sep 27;13:90. doi: 10.1186/1471-2350-13-90 (PMC3570299; doi:10.1186/1471-2350-13-90)
Supplement: Additional file 3 — Table S3. Association of SNPs in fasting glucose and type 2 diabetes-related genes in children with IQ at age 8. [file 1471-2350-13-90-S3.doc]

**Additional table 3.** Association of SNPs in fasting glucose and type 2 diabetes-related genes in children with IQ at age 8.

| **gene** | **dbSNP id** | **mean difference in IQ**  **per minor allele (95% CI)** | **p-value** | **N** |
| --- | --- | --- | --- | --- |
| *ADAMTS9* | rs4607103 | 0.21 (-0.54, 0.96) | 0.58 | 4901 |
| *ADCY5* | rs2877716 | 0.54 (-0.20, 1.28) | 0.15 | 4806 |
| *ADIPOQ* | rs1501399 | 0.32 (-0.41, 1.04) | 0.39 | 4952 |
| *ADIPOQ* | rs17300539 | -1.06 (-2.19, 0.07) | 0.07 | 4892 |
| *ADIPOQ* | rs266729 | -0.32 (-1.06, 0.42) | 0.40 | 4957 |
| *ADRA2A* | rs10885122 | -0.72 (-1.63, 0.19) | 0.12 | 5617 |
| *C2CD4B* | rs11071657 | 0.13 (-0.49, 0.75) | 0.68 | 5635 |
| *CDC123/CAMK1D* | rs12779790 | 0.47 (-0.35, 1.30) | 0.26 | 4883 |
| *CDKAL1* | rs10946398 | 0.20 (-0.49, 0.89) | 0.57 | 4891 |
| *CDKN2A/2B* | rs10811661 | -1.02 (-1.87, -0.18) | 0.02 | 4928 |
| *COX2* | rs20417 | 0.77 (-0.07, 1.61) | 0.07 | 5574 |
| *CRY2* | rs1160592 | -0.03 (-0.57, 0.63) | 0.92 | 5625 |
| *DGKB/TMEM195* | rs2191349 | 0.15 (-0.50, 0.79) | 0.66 | 4906 |
| *FADS1* | rs174550 | 0.08 (-0.57, 0.72) | 0.81 | 5613 |
| *FTO* | rs9939609 | -0.28 (-0.94, 0.38) | 0.40 | 4867 |
| *G6PC2* | rs560887 | 0.14 (-0.56, 0.85) | 0.69 | 4922 |
| *GCK* | rs1799884 | 0.17 (-0.64, 0.99) | 0.68 | 5112 |
| *GCKR* | rs780094 | -0.07 (-0.73, 0.59) | 0.83 | 4922 |
| *GLIS3* | rs7034200 | 0.57 (-0.04, 1.17) | 0.07 | 5579 |
| *HHEX-IDE* | rs1111875 | -0.07 (-0.72, 0.58) | 0.83 | 4933 |
| *HNFB1* | rs757210 | -0.08 (-0.73, 0.57) | 0.81 | 4855 |
| *IGF2BP2* | rs4402690 | 0.01 (-0.68, 0.71) | 0.97 | 4938 |
| *JAZF1* | rs864745 | -0.11 (-0.76, 0.53) | 0.73 | 4879 |
| *KCNJ11* | rs5219 | -0.04 (-0.70, 0.62) | 0.91 | 4915 |
| *KCNQ1* | rs2237892 | 1.25 (-0.12, 2.62) | 0.07 | 4892 |
| *KCNQ1* | rs2237895 | -0.19 (-0.84, 0.45) | 0.56 | 4914 |
| *MADD* | rs7944584 | 0.43 (-0.25, 1.11) | 0.25 | 5623 |
| *MTNR1B* | rs10830963 | -0.18 (-0.89, 0.54) | 0.63 | 4922 |
| *NOTCH2* | rs10923931 | 0.43 (-0.61, 1.47) | 0.42 | 4924 |
| *PPARG* | rs1801282 | -1.18 (-2.20, -0.16) | 0.02 | 4898 |
| *PROX1* | rs340874 | 0.11 (-0.51, 0.72) | 0.74 | 5626 |
| *SLC2A2* | rs11920090 | -0.14 (-1.04, 0.77) | 0.77 | 5623 |
| *SLC30A8* | rs13266634 | 0.15 (-0.54, 0.85) | 0.67 | 4877 |
| *TCF7L2* | rs12255372 | -0.19 (-0.89, 0.50) | 0.59 | 5010 |
| *TCF7L2* | rs7903146 | 0.02 (-0.67, 0.72) | 0.94 | 4806 |
| *THADA* | rs7578597 | 0.17 (-0.84, 1.17) | 0.74 | 4920 |
| *TSPAN8-LGR5* | rs7961581 | 0.76 (0.05, 1.48) | 0.04 | 4881 |
| *WFS1* | rs10010131 | 0.32 (-0.33, 0.97) | 0.34 | 4947 |
