# Supplementary material for: Maternal and offspring fasting glucose and type 2 diabetes-associated genetic variants and cognitive function at age 8: a Mendelian randomization study in the Avon Longitudinal Study of Parents and Children
Source: BMC Med Genet. 2012 Sep 27;13:90. doi: 10.1186/1471-2350-13-90 (PMC3570299; doi:10.1186/1471-2350-13-90)
Supplement: Additional file 4 — Table S4. Association of SNPs in fasting glucose and type 2 diabetes-related genes in children with IQ at age 8, adjusted for population stratification. [file 1471-2350-13-90-S4.doc]

**Additional Table 4.** Association of SNPs in fasting glucose and type 2 diabetes-related genes in children with IQ at age 8, adjusted for population stratification.

| **gene** | **dbSNP id** | **mean difference in IQ**  **per minor allele (95% CI)** | **p-value** | **N** |
| --- | --- | --- | --- | --- |
| *ADAMTS9* | rs4607103 | 0.08 (-0.73, 0.90) | 0.84 | 4238 |
| *ADCY5* | rs2877716 | 0.68 (-0.13, 1.48) | 0.10 | 4160 |
| *ADIPOQ* | rs1501399 | 0.30 (-0.48, 1.08) | 0.45 | 4277 |
| *ADIPOQ* | rs17300539 | -0.96 (-2.18, 0.25) | 0.12 | 4231 |
| *ADIPOQ* | rs266729 | -0.33 (-1.13, 0.47) | 0.42 | 4286 |
| *ADRA2A* | rs10885122 | -0.95 (-1.92, 0.03) | 0.06 | 4953 |
| *C2CD4B* | rs11071657 | 0.12 (-0.54, 0.78) | 0.71 | 4973 |
| *CDC123/CAMK1D* | rs12779790 | 0.59 (-0.30, 1.48) | 0.19 | 4227 |
| *CDKAL1* | rs10946398 | 0.13 (-0.62, 0.89) | 0.73 | 4227 |
| *CDKN2A/2B* | rs10811661 | -1.02 (-1.94, -0.10) | 0.03 | 4261 |
| *COX2* | rs20417 | 0.92 (0.02, 1.82) | 0.05 | 4918 |
| *CRY2* | rs1160592 | -0.12 (-0.76, 0.52) | 0.71 | 4968 |
| *DGKB/TMEM195* | rs2191349 | 0.27 (-0.43, 0.96) | 0.45 | 4238 |
| *FADS1* | rs174550 | 0.02 (-0.67, 0.70) | 0.96 | 4957 |
| *FTO* | rs9939609 | -0.47 (-1.19, 0.25) | 0.20 | 4217 |
| *G6PC2* | rs560887 | 0.17 (-0.59, 0.93) | 0.66 | 4257 |
| *GCK* | rs1799884 | -0.19 (-1.08, 0.70) | 0.68 | 4349 |
| *GCKR* | rs780094 | -0.15 (-0.87, 0.56) | 0.68 | 4255 |
| *GLIS3* | rs7034200 | 0.44 (-0.20, 1.09) | 0.18 | 4926 |
| *HHEX-IDE* | rs1111875 | -0.11 (-0.81, 0.60) | 0.77 | 4263 |
| *HNFB1* | rs757210 | -0.08 (-0.78, 0.62) | 0.83 | 4199 |
| *IGF2BP2* | rs4402690 | 0.28 (-0.47, 1.03) | 0.46 | 4271 |
| *JAZF1* | rs864745 | -0.06 (-0.75, 0.64) | 0.87 | 4221 |
| *KCNJ11* | rs5219 | 0.35 (-0.36, 1.06) | 0.34 | 4251 |
| *KCNQ1* | rs2237892 | 1.64 (0.16, 3.13) | 0.03 | 4230 |
| *KCNQ1* | rs2237895 | -0.21 (-0.90, 0.47) | 0.54 | 4256 |
| *MADD* | rs7944584 | 0.44 (-0.28, 1.17) | 0.23 | 4962 |
| *MTNR1B* | rs10830963 | -0.18 (-0.96, 0.60) | 0.66 | 4253 |
| *NOTCH2* | rs10923931 | 0.70 (-0.42, 1.82) | 0.22 | 4258 |
| *PPARG* | rs1801282 | -1.35 (-2.44, -0.26) | 0.02 | 4241 |
| *PROX1* | rs340874 | -0.07 (-0.73, 0.59) | 0.84 | 4961 |
| *SLC2A2* | rs11920090 | 0.02 (-0.95, 0.99) | 0.97 | 4963 |
| *SLC30A8* | rs13266634 | 0.20 (-0.55, 0.95) | 0.61 | 4221 |
| *TCF7L2* | rs12255372 | -0.13 (-0.89, 0.62) | 0.73 | 4313 |
| *TCF7L2* | rs7903146 | 0.11 (-0.65, 0.86) | 0.79 | 4142 |
| *THADA* | rs7578597 | -0.01 (-1.10, 1.08) | 0.99 | 4250 |
| *TSPAN8-LGR5* | rs7961581 | 0.59 (-0.19, 1.36) | 0.14 | 4217 |
| *WFS1* | rs10010131 | 0.17 (-0.53, 0.88) | 0.63 | 4276 |
