# Supplementary material for: Maternal and offspring fasting glucose and type 2 diabetes-associated genetic variants and cognitive function at age 8: a Mendelian randomization study in the Avon Longitudinal Study of Parents and Children
Source: BMC Med Genet. 2012 Sep 27;13:90. doi: 10.1186/1471-2350-13-90 (PMC3570299; doi:10.1186/1471-2350-13-90)
Supplement: Additional file 5 — Table S5. Association of maternal SNPs in fasting glucose and type 2 diabetes-related genes with offspring IQ at age 8. [file 1471-2350-13-90-S5.doc]

**Supplementary Table 5.** Association of maternal SNPs in fasting glucose and type 2 diabetes-related genes with offspring IQ at age 8.

| **gene** | **dbSNP id** | **mean difference in IQ**  **per minor allele (95% CI)** | **p-value** | **N** |
| --- | --- | --- | --- | --- |
| *ADAMTS9* | rs4607103 | 0.32 (-0.52, 1.16) | 0.45 | 3953 |
| *ADCY5* | rs2877716 | -0.29 (-1.10, 0.52) | 0.49 | 3950 |
| *ADRA2A* | rs10885122 | -0.66 (-1.82, 0.49) | 0.26 | 3537 |
| *C2CD4B* | rs11071657 | 0.46 (-0.32, 1.25) | 0.19 | 3519 |
| *CDC123/CAMK1D* | rs12779790 | -0.92 (-2.44, 0.61) | 0.24 | 1503 |
| *CDKAL1* | rs10946398 | -0.10 (-0.88, 0.68) | 0.80 | 3956 |
| *CDKN2A/2B* | rs10811661 | -0.92 (-1.87, -0.02) | 0.06 | 3979 |
| *COX2* | rs20417 | 0.55 (-0.51, 1.62) | 0.31 | 3517 |
| *CRY2* | rs1160592 | 0.08 (-0.69, 0.86) | 0.84 | 3536 |
| *DGKB/TMEM195* | rs2191349 | -0.42 (-1.15, 0.31) | 0.26 | 3945 |
| *FADS1* | rs174550 | 0.46 (-0.35, 1.27) | 0.27 | 3533 |
| *FTO* | rs9939609 | -0.24 (-0.98, 0.50) | 0.52 | 3976 |
| *G6PC2* | rs560887 | -0.07 (-0.87, 0.73) | 0.87 | 3916 |
| *GCK* | rs1799884 | -0.48 (-1.43, 0.47) | 0.32 | 3953 |
| *GCKR* | rs780094 | 0.18 (-0.56, 0.92) | 0.64 | 3972 |
| *GLIS3* | rs7034200 | 0.77 (-0.01, 1.54) | 0.05 | 3482 |
| *HHEX-IDE* | rs1111875 | -0.36 (-1.10, 0.37) | 0.33 | 3976 |
| *HNFB1* | rs757210 | -0.17 (-0.93, 0.58) | 0.66 | 3843 |
| *IGF2BP2* | rs4402690 | -0.38 (-1.16, 0.41) | 0.34 | 3979 |
| *JAZF1* | rs864745 | -0.92 (-1.64, -0.19) | 0.01 | 3968 |
| *KCNJ11* | rs5219 | 0.42 (-0.34, 1.18) | 0.28 | 3934 |
| *KCNQ1* | rs2237892 | -0.62 (-2.16, 0.91) | 0.42 | 3930 |
| *KCNQ1* | rs2237895 | -0.06 (-0.79, 0.68) | 0.88 | 3935 |
| *MADD* | rs7944584 | 0.31 (-0.54, 1.17) | 0.47 | 3519 |
| *MTNR1B* | rs10830963 | -0.45 (-1.26, 0.37) | 0.28 | 3947 |
| *NOTCH2* | rs10923931 | -0.98 (-2.15, 0.18) | 0.10 | 3995 |
| *PPARG* | rs1801282 | -0.96 (-2.09, 0.17) | 0.10 | 3979 |
| *PROX1* | rs340874 | 0.96 (0.17, 1.76) | 0.02 | 3523 |
| *SLC2A2* | rs11920090 | 0.50 (-0.71, 1.70) | 0.42 | 3540 |
| *SLC30A8* | rs13266634 | 0.18 (-0.61, 0.97) | 0.65 | 3965 |
| *TCF7L2* | rs12255372 | 0.29 (-0.51, 1.08) | 0.48 | 4006 |
| *TCF7L2* | rs7903146 | 0.13 (-0.67, 0.94) | 0.74 | 3840 |
| *THADA* | rs7578597 | 0.11 (-1.04, 1.26) | 0.85 | 3975 |
| *TSPAN8-LGR5* | rs7961581 | 0.12 (-0.68, 0.93) | 0.76 | 3948 |
| *WFS1* | rs10010131 | 0.60 (-0.14, 1.34) | 0.11 | 3953 |
