# Supplementary material for: Maternal and offspring fasting glucose and type 2 diabetes-associated genetic variants and cognitive function at age 8: a Mendelian randomization study in the Avon Longitudinal Study of Parents and Children
Source: BMC Med Genet. 2012 Sep 27;13:90. doi: 10.1186/1471-2350-13-90 (PMC3570299; doi:10.1186/1471-2350-13-90)
Supplement: Additional file 6 — Table S6. Association of maternal SNPs in fasting glucose and type 2 diabetes-related genes with offspring IQ at age 8, adjusted for offspring genotype and population stratification. [file 1471-2350-13-90-S6.doc]

**Additional Table 6.** Association of maternal SNPs in fasting glucose and type 2 diabetes-related genes with offspring IQ at age 8, adjusted for offspring genotype and population stratification.

| **gene** | **dbSNP id** | **mean difference in IQ**  **per minor allele (95% CI)** | **p-value** | **N** |
| --- | --- | --- | --- | --- |
| *ADAMTS9* | rs4607103 | -0.40 (-1.63, 0.84) | 0.53 | 2488 |
| *ADCY5* | rs2877716 | -0.63 (-1.82, 0.55) | 0.29 | 2454 |
| *ADRA2A* | rs10885122 | -0.79 (-2.36, 0.78) | 0.32 | 2510 |
| *C2CD4B* | rs11071657 | -0.24 (-1.32, 0.85) | 0.67 | 2512 |
| *CDC123/CAMK1D* | rs12779790 | -2.18 (-4.42, 0.07) | 0.06 | 945 |
| *CDKAL1* | rs10946398 | 0.51 (-0.62, 1.64) | 0.37 | 2496 |
| *CDKN2A/2B* | rs10811661 | -1.09 (-2.48, 0.30) | 0.12 | 2526 |
| *COX2* | rs20417 | -0.52 (-1.94, 0.90) | 0.47 | 2475 |
| *CRY2* | rs1160592 | 0.52 (-0.54, 1.58) | 0.34 | 2510 |
| *DGKB/TMEM195* | rs2191349 | -0.79 (-1.84, 0.26) | 0.14 | 2499 |
| *FADS1* | rs174550 | 0.41 (-0.70, 1.51) | 0.47 | 2506 |
| *FTO* | rs9939609 | -0.48 (-1.55, 0.60) | 0.39 | 2497 |
| *G6PC2* | rs560887 | -1.34 (-2.47, -0.20) | 0.02 | 2488 |
| *GCK* | rs1799884 | -0.91 (-2.24, 0.43) | 0.18 | 2590 |
| *GCKR* | rs780094 | 0.20 (-0.87, 1.28) | 0.71 | 2514 |
| *GLIS3* | rs7034200 | 0.61 (-0.44, 1.67) | 0.25 | 2459 |
| *HHEX-IDE* | rs1111875 | -0.39 (-1.45, 0.68) | 0.48 | 2530 |
| *HNFB1* | rs757210 | -0.22 (-1.31, 0.86) | 0.69 | 2430 |
| *IGF2BP2* | rs4402690 | -0.73 (-1.85, 0.40) | 0.21 | 2540 |
| *JAZF1* | rs864745 | -1.26 (-2.30, -0.21) | 0.02 | 2494 |
| *KCNJ11* | rs5219 | 0.95 (-0.14, 2.04) | 0.09 | 2500 |
| *KCNQ1* | rs2237892 | -1.31 (-3.48, 0.86) | 0.24 | 2458 |
| *KCNQ1* | rs2237895 | 0.75 (-0.31, 1.82) | 0.17 | 2497 |
| *MADD* | rs7944584 | 1.00 (-0.15, 2.16) | 0.09 | 2500 |
| *MTNR1B* | rs10830963 | -0.32 (-1.49, 0.86) | 0.60 | 2506 |
| *NOTCH2* | rs10923931 | -1.25 (-2.98, 0.48) | 0.14 | 2529 |
| *PPARG* | rs1801282 | -1.04 (-2.66, 0.59) | 0.21 | 2498 |
| *PROX1* | rs340874 | 1.30 (0.24, 2.35) | 0.02 | 2499 |
| *SLC2A2* | rs11920090 | 1.15 (-0.50, 2.79) | 0.17 | 2517 |
| *SLC30A8* | rs13266634 | 0.04 (-1.10, 1.19) | 0.94 | 2488 |
| *TCF7L2* | rs12255372 | 0.77 (-0.38, 1.91) | 0.19 | 2577 |
| *TCF7L2* | rs7903146 | 0.71 (-0.47, 1.88) | 0.24 | 2380 |
| *THADA* | rs7578597 | 0.26 (-1.43, 1.95) | 0.76 | 2510 |
| *TSPAN8-LGR5* | rs7961581 | 0.27 (-0.89, 1.44) | 0.65 | 2491 |
| *WFS1* | rs10010131 | 1.38 (0.31, 2.44) | 0.01 | 2514 |
